# Mutational analysis of a *Drosophila* neuroblast enhancer governing *nubbin* expression during CNS development

**DOI:** 10.1002/dvg.23237

**Published:** 2018-08-18

**Authors:** Jermaine Ross, Alexander Kuzin, Thomas Brody, Ward F. Odenwald

**Affiliations:** ^1^ Neural Cell‐Fate Determinants Section NINDS, NIH Bethesda Maryland

**Keywords:** CNS, *Drosophila*, enhancers, transcriptional regulation

## Abstract

While developmental studies of *Drosophila* neural stem cell lineages have identified transcription factors (TFs) important to cell identity decisions, currently only an incomplete understanding exists of the *cis*‐regulatory elements that control the dynamic expression of these TFs. Our previous studies have identified multiple enhancers that regulate the POU‐domain TF paralogs *nubbin* and *pdm‐2* genes. Evolutionary comparative analysis of these enhancers reveals that they each contain multiple conserved sequence blocks (CSBs) that span TF DNA‐binding sites for known regulators of neuroblast (NB) gene expression in addition to novel sequences. This study functionally analyzes the conserved DNA sequence elements within a NB enhancer located within the *nubbin* gene and highlights a high level of complexity underlying enhancer structure. Mutational analysis has revealed CSBs that are important for enhancer activation and silencing in the developing CNS. We have also observed that adjusting the number and relative positions of the TF binding sites within these CSBs alters enhancer function.

## 1 INTRODUCTION

Enhancers consist of *cis*‐acting DNA elements that control the spatial and temporal aspects of gene expression (reviewed by Epstein, 2009). Although previous studies have shown that enhancers contain clusters of transcription factor (TF) binding sites within blocks of conserved sequences (Berman et al., 2004; Brody et al., 2012; Davidson & Erwin, 2006; Swanson, Evans, & Barolo, 2010), it is not yet well understood just how the distribution of these sites provides a basis for combinatorial logic of enhancer function. One entry point into deciphering the rules that govern an enhancer's ability to direct gene expression is to manipulate the internal organization of enhancers, that is, position, frequency, and/or order of functionally relevant sequences and examine the effects on *cis*‐regulatory behavior. For example, altering the TF binding sites and other sequences within the *sparkling* enhancer of *shaven* (dPax), a gene that encodes a key regulator of photoreceptor fate specification in the developing *Drosophila* retina, switched the specificity of the enhancer from cone to rod photoreceptors (Swanson et al., 2010). A second study has shown that an enhancer of the *Drosophila Suppressor of Hairless* gene is composed of overlapping elements termed submodules that can function independently to activate enhancer activity (Liu & Posakony, 2014). In addition, a recent study of a notochord enhancer structure in *Ciona* points to the importance of TF binding site affinity and arrangement in conferring tissue specificity on enhancer function (Farley, Olson, Zhang, Rokhsar, & Levine, 2016; reviewed by Barolo, 2016; Crocker, Noon, & Stern, 2016). The presence of repeat sequence motifs in these enhancers, and their conserved positioning, points to the necessity of considering binding site position and TF avidity (Levo & Segal, 2014; Sayal, Dresch, Pushel, Taylor, & Arnosti, 2016).

*nubbin* (*nub*) and its closely linked paralog *pdm‐2* encode POU homeodomain TFs are known for their role in neurogenesis (Billin, Cockerill, & Poole, 1991; Dick, Yang, Yeo, & Chia, 1991; Lloyd & Sakonju, 1991). During embryonic CNS development, the sequential NB expression of the TFs Hunchback→ Krüppel→ Nubbin and Pdm‐2→ Castor→ Grainyhead coordinates specification of neuronal temporal identity (reviewed by Brody & Odenwald, 2002, 2005; Chai, Liu, Chia, & Cai, 2013; Kuzin et al., 2012; Syed, Mark, & Doe, 2017). Loss of any of these TFs, including *nubbin* and *pdm‐2*, triggers abnormal CNS development and consequently embryonic lethality (Grosskortenhaus, Pearson, Marusich, & Doe, 2005; Kohwi & Doe, 2013; Tran & Doe, 2008; Yang, Yeo, Dick, & Chia, 1993; Yeo et al., 1995). The *pdm* genes are expressed in overlapping but non‐identical patterns within intermediate neuroblast sublineages. Together, Hb and Cas silence *pdm* expression in early and late forming sublineages, respectively, thereby limiting *pdm* expression to intermediate sublineages (reviewed by Brody & Odenwald, 2002; Syed et al., 2017). One of the major questions concerning the sequential expression of these TFs is the regulatory basis of their temporal gene expression. Experimental results suggest that the network is regulated by repression of the TFs. This model has been further elaborated to include both activation and feedback repression to achieve temporal gene expression (Nakajima, 2010).

The aim of this study is to understand the regulation of *nubbin* NB expression in terms of its conserved *cis*‐regulatory sequences. Previous work with a 3.2 Kb fragment that includes a NB enhancer showed that loss of *cas* function, acting through Cas target sequences, resulted in ectopic activation of *pdm* expression during embryonic lineage development (Kambadur et al., 1998). A subsequent study identified and delimited the NB enhancers within the *pdm* locus (Ross, Kuzin, Brody, & Odenwald, 2015). One of these enhancers lies within a *nub* intron, denoted as *nub‐46*. Phylogenetic footprinting of the *nub‐46* enhancer reveals that it contains multiple conserved sequence blocks (CSBs). This study describes the functional characterization of the *nub‐46* NB enhancer in terms of its conserved sequences. Truncation analysis of the CSB cluster was used to delimit core elements that are required for embryonic enhancer expression. We examined the roles of each of the *nub‐46* CSBs using deletions and sequence rearrangement to resolve sequences that are required for temporal and spatial regulation. Within the core element, we identified two consensus Cas binding sites. Our functional analysis has revealed (a) that the Cas binding sites in *nub‐46* are the targets of Cas mediated repression; (b) that CSBs containing repeat sequences are not required for enhancer activation; and (c) that novel non‐repeated conserved sequences are essential for enhancer activity.

## 2 MATERIALS AND METHODS

### 2.1 Comparative genomics

The phylogenetic comparative analysis of the *nub‐46* enhancer was performed using the *EvoPrinterHD* program (http://evoprinter.ninds.nih.gov/)), a program providing alignment of 12 sequenced *Drosophila* genomes (Odenwald, Rasband, Kuzin, & Brody, 2005; Yavatkar et al., 2008). Instructions for using the *EvoPrinterHD* comparative tool are provided on the *EvoPrinter* website.

### 2.2 Enhancer‐reporter transgene vector

A modified pCa4B vector was employed in these studies (Brody et al., 2012). The pCa4B vector was modified to include the following features from the pHStinger vector (Barolo, Castro, & Posakony, 2004): the pHStinger polylinker (replacing the pCa4B polylinker), a minimal Heat shock protein 70 (Hsp70) promoter driving a GFP or RFP reporter gene, and gypsy chromatin insulators to block influence of flanking enhancers that would otherwise modify reporter expression via enhancer trap effects. The vector also contains bacterial attachment (attB) sites for its targeted chromosomal insertion (Groth & Calos, 2004). The site‐specific integration vector was selected to ensure that all of the enhancer‐reporter constructs were inserted in the same chromosomal environment. In addition to the gypsy chromatin insulators, the nonrandom integration afforded by the PhiC31 integration further reduces integration variability on enhancer function. Integration of the pCa4B vector is facilitated by a serine integrase, phage PhiC31, which mediates recombination between vector attB sites and genomic attP sites (Groth & Calos, 2004).

### 2.3 Transgene constructs

*nub‐46* enhancer DNA fragment was cloned from wild‐type genomic DNA using standard PCR method (*nub‐46* 5′‐primer is TATTAGGCAACTGTCCTCTGCC and *nub‐46* 3′‐primer is ACTGAACAGGGTAGCTATTCGG). PCR products were analyzed using gel electrophoresis and were purified by a Qiagen QIAquick Gel Extraction Kit. Purified PCR products were inserted into the Invitrogen pCRII‐TOPO TA vectors. For CSB deletions and rearrangements, we employed the Invitrogen GeneArt Gene Service to generate mutated *nub‐46* enhancers. Verified sequences were inserted into the modified pCa4B vector (details are available upon request).

### 2.4 Generation of transgenic fly lines

Transgenes were injected into either VK1 (insertion site on chromosome 2R, 59D3) or attP2 (insertion site on chromosome 3L, 68A4) embryos by Rainbow Transgenic Flies, and at least two independent transformant lines for each construct were generated. Standard genetic crosses were performed to generate homozygous transgenic fly lines. Fly lines are maintained at 18 °C using standard husbandry procedures (Ashburner, 1989).

### 2.5 In situ hybridization and immunohistochemistry

Embryo collections and fixations of at least two independent lines per construct were performed according to procedures previously described (Tomancak et al., 2002). For in situ hybridizations, mRNA probes were generated from a PCR amplified GFP ORF. Roche DIG RNA Labeling Mix protocol was used, and staining was visualized using anti‐DIG Fab fragments coupled to alkaline phosphatase (1:2,000, Roche). Whole‐mount or filleted embryos were photographed using a Nikon Optiphot microscope (10X objective lens). Embryo developmental stages were determined based on morphological features previously described (Campos‐Ortega, 1995). Immunolabeling experiments used anti‐Cas rabbit antibodies (1:500) and anti‐GFP chicken antibodies (1:500, Chemicon). Secondary antibodies included anti‐chicken Alexa 488 (1:1,000, Invitrogen), anti‐rabbit Alexa 633 (1:1,000, Invitrogen). After immunolabeling, embryos were examined for GFP and Cas expression via serial optical sections that were photographed at 1 μm intervals using a Zeiss LSM 510 confocal microscope. Detailed protocols are available upon request.

## 3 RESULTS AND DISCUSSION

### 3.1 *nub‐46* sequence conservation and embryonic cis‐regulatory dynamics

**Figure 1:**
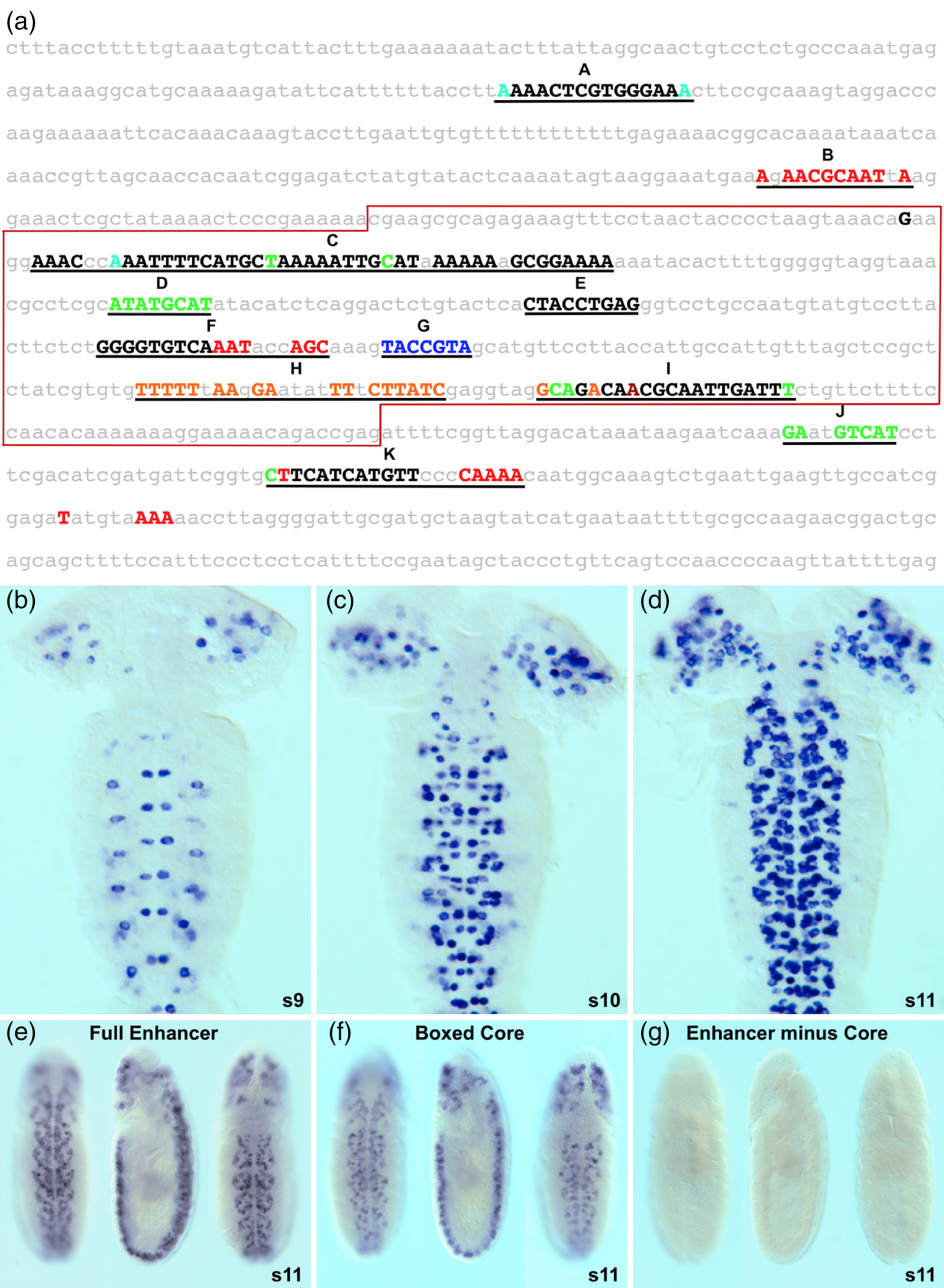
*nub‐46* enhancer evolutionary conservation, embryonic *cis*‐regulatory dynamics, and identification of essential core elements. (a) Relaxed *EvoPrint* of the *D. melanogaster nub‐46* enhancer genomic region (936 bp) identifies 11 conserved sequence blocks (labeled A through K). The readout is a composite of superimposed pairwise alignments between the *D. melanogaster* reference sequence and 11 other *Drosophila* species that together represent ~200 million years of cumulative evolutionary divergence. Black uppercase bases are conserved in all species, while colored bases are conserved in all but one of the color‐coded *Drosophila* species: *D. sechellia*, *D. simulans*, *D. yakuba*, *D. erecta*, *D. ananassae*, *D. pseudoobscura*, *D. persimilis*, *D. willistoni*, *D. virilis*, *D. grimshawi*, *or D. mojavensis*. Lowercase gray‐colored *D. melanogaster* bases are less‐conserved and differ in two or more of the 11 species. (b–d) Flattened embryo fillets (stages 9, 10, and 11; anterior up) highlight *nub‐46* enhancer/reporter transgene mRNA expression during intermediate stages of CNS neurogenesis. Note the progressive activation of the *nub‐46* enhancer in both the developing cephalic lobe and ventral cord neuroblasts. (e–g) Truncation and deletion analysis of the *nub‐46* enhancer identifies core sequences that are essential for its *cis*‐regulatory activity: enhancer/reporter mRNA expression patterns in whole‐mount stage 11 embryos (ventral, lateral, and dorsal views; anterior up). (e) Full *nub‐46* enhancer. (f) Boxed region minus flanks (shown in panel a). (g) Full enhancer with boxed region deleted

Our previous enhancer‐reporter transgene survey identified an enhancer (denoted as *nub‐46*) that recapitulated *nub* expression during embryonic cephalic lobe and VNC NB lineage development (Ross et al., 2015). As an initial step to functionally characterize the *nub‐46* enhancer, we identified its conserved sequence blocks by comparative evolutionary analysis using 12 *Drosophila* species, including *D. melanogaster*, *D. simulans*, *D. sechellia*, *D. yakuba*, *D. erecta*, *D. ananassae*, *D. persimilis*, *D. pseudoobscura*, *D. willistoni*, *D. virilis*, *D. mojavensis*, and *D. grimshawi*. Our analysis revealed that *nub‐46* is made up of 11 CSBs (Figure 1a). While many of its conserved elements are novel, we identified a CSB, denoted as “C” (Figure 1a), containing two adjacent 9‐mer sequences (TAAAAATTG and CATAAAAAA) that correspond to the DNA‐binding site motifs for Cas (Kambadur et al., 1998).

The *nub‐46* enhancer‐reporter transgene expression is dynamic during embryonic CNS development. We observed transient *nub‐46* activation at the cellular blastoderm stage (data not shown), followed by progressive NB reactivation during embryonic neurogenesis (Figure 1b–d). At stage 9, *nub‐46* regulates transgene reporter expression in several NBs per ventral cord hemisegment, and enhancer activity is detected in a subset of cephalic lobe NBs (Figure 1b). Later in CNS development enhancer/reporter expression is detected in additional cephalic lobe and ventral cord NB lineages (Figure 1c,d). After embryonic stage 13, *nub‐46* cis‐regulatory activity is downregulated in both the brain and ventral cord (data not shown).

### 3.2 Identification of the *nub‐46* core enhancer

To delimit the boundaries of the *nub‐46* enhancer, we generated both 5′ and 3′ deletions of the full *nub‐46* enhancer CSB cluster (Ross et al., 2015) and examined the in vivo cis‐regulatory activity of these truncated fragments via enhancer‐reporter transgenes. This analysis revealed that the centrally located CSBs (Figure 1a, “C” through “I”) were sufficient for embryonic CNS expression (see Figure 1f). However, compared to the full‐length enhancer, we observed a reduced enhancer/reporter activity for the core that contains elements “C” through “I” (compare Figure 1e,f). These findings demonstrate that the core fragment consists of activator and repressor sequences required for its wild‐type spatial and temporal regulatory dynamics.

### 3.3 Castor regulates the *nub‐46* enhancer during embryonic neurogenesis

**Figure 2:**
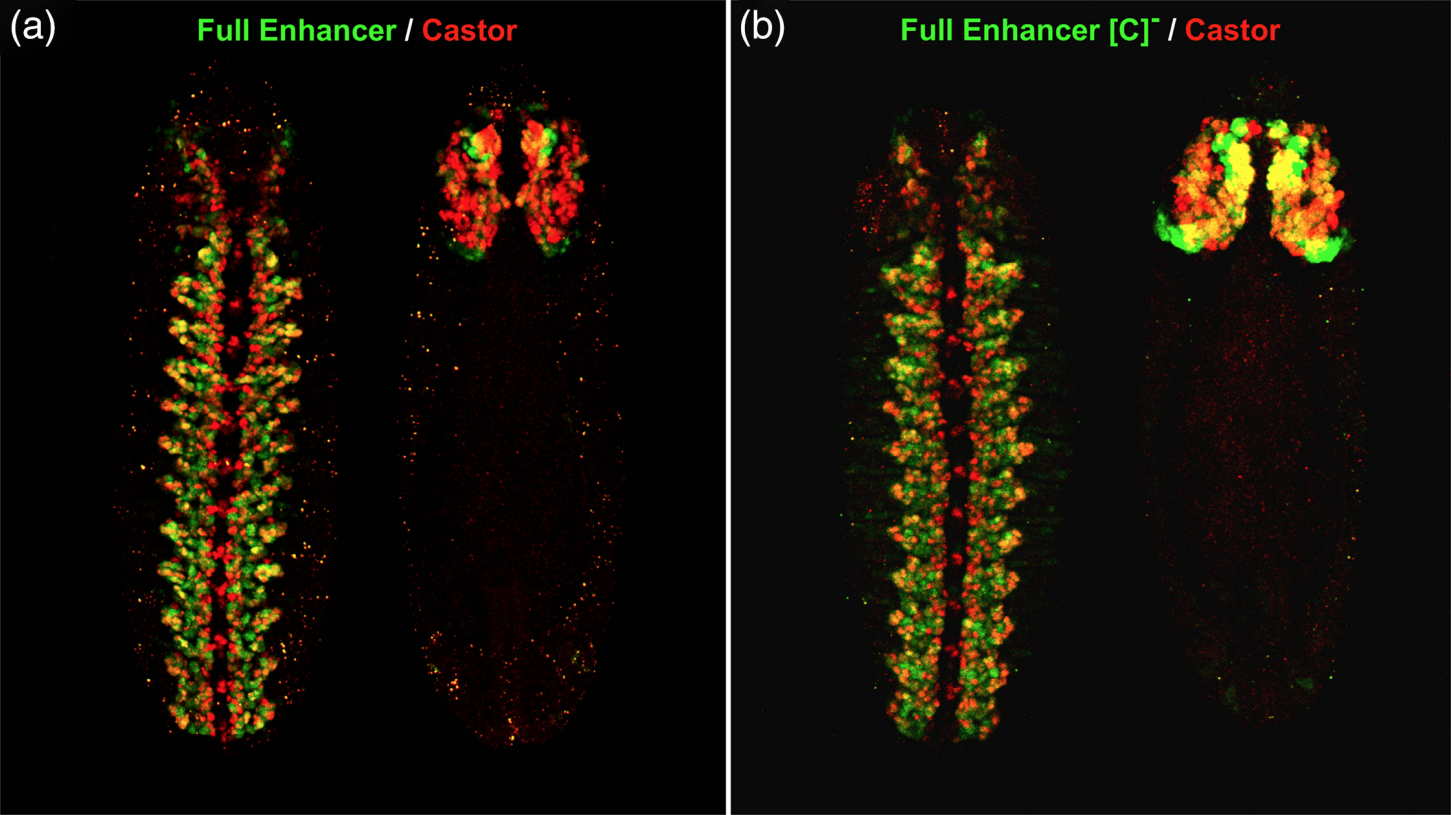
*nub‐46* enhancer conserved sequence block “C” functions as a *cis*‐regulatory repressor element to restrict cephalic lobe enhancer activity during stage 12 CNS development. Whole‐mount confocal views of co‐immunostained stage 13 embryos (ventral and dorsal views left to right; anterior up) showing: (a) full *nub‐46* enhancer/reporter transgene expression in green and endogenous Castor (Cas) protein in red; and (b) full enhancer without the conserved sequence block (CSB) “C” (see Figure 1a) in green and Cas expression in red. During stage 13, *nub‐46* enhancer activity is detected in a limited subset of Cas positive ventral cord and cephalic lobe neural lineages. Deletion of the *nub‐46* enhancer CSB “C” results in ectopic enhancer activity, most notably, in a subset of Cas expressing cephalic lobe neuroblasts

Given that Cas is a negative regulator of *pdm* gene expression in embryonic NBs, we predicted that the putative Cas DNA‐binding motifs within *nub‐46* are required to deactivate enhancer activity. Expression of *nub‐46* enhancer activity partially overlaps endogenous Cas protein expression in stage 13 embryos (Figure 2a). To determine whether the putative Cas binding‐motifs function as Cas binding sites, we examined the regulatory activity of a *nub‐46* deletion that lacks a 40 bp conserved region containing the two Cas motifs (Figure 2b). Deletion of the Cas DNA‐binding sites triggers ectopic enhancer activity in the cephalic lobes during stage 13 (Figure 2b), suggesting that the “C” CSB functions as a repressor element during cephalic lobe development. Interestingly, we did not observe significant ectopic enhancer activity in the developing VNC. Therefore, removal of the *nub‐46* “C” CSB does not completely account for the repressive action of Cas on the *nub‐46* enhancer, especially in the VNC, and other direct or indirect effects of Cas action on the *nub* should be considered.

### 3.4 Deletion analysis of the core *nub‐46* CSBs

**Figure 3:**
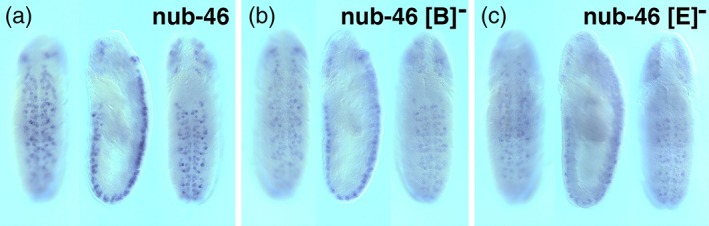
Deletion analysis of the *nub‐46* enhancer reveals CSBs whose removal does not significantly alter embryonic enhancer activity. Transgene reporter mRNA expression patterns in whole‐mount stage 11 embryos (ventral and dorsal views; anterior up). (a) Intact *nub‐46* enhancer, (b) deletion of the “B” CSB, (c) deletion of the “E” CSB (see Figure 1a for sequences)

While the “C” element may contain repressor DNA‐binding sites, it remained unknown how the *nub‐46* enhancer is activated in the embryonic CNS. To address this question, we further examined the effects of internal deletions within the *nub‐46* enhancer. Each of the 10 remaining CSBs illustrated in Figure 1 were individually removed. Enhancers with these individual deletions were tested in two independent transgenic lines. The wild type control enhancer activity was tested under the same conditions and at the same time as the deletion mutants. We observed that *nub‐46* variants lacking either the “B**”** (AGAACGCAAT) element or “E**”** (CTACCTGAG) element displayed only a modest reduction in enhancer activity compared to the wild‐type (Figure 3). Surprisingly, we found that singular removal of other CSBs had only subtle effects on enhancer activity during embryonic NB lineage development (data not shown), suggesting that these CSBs may be either required at later time points or are functionally redundant.

### 3.5 *nub*‐46 enhancer contains multiple activator sequences within its conserved core elements

**Figure 4:**
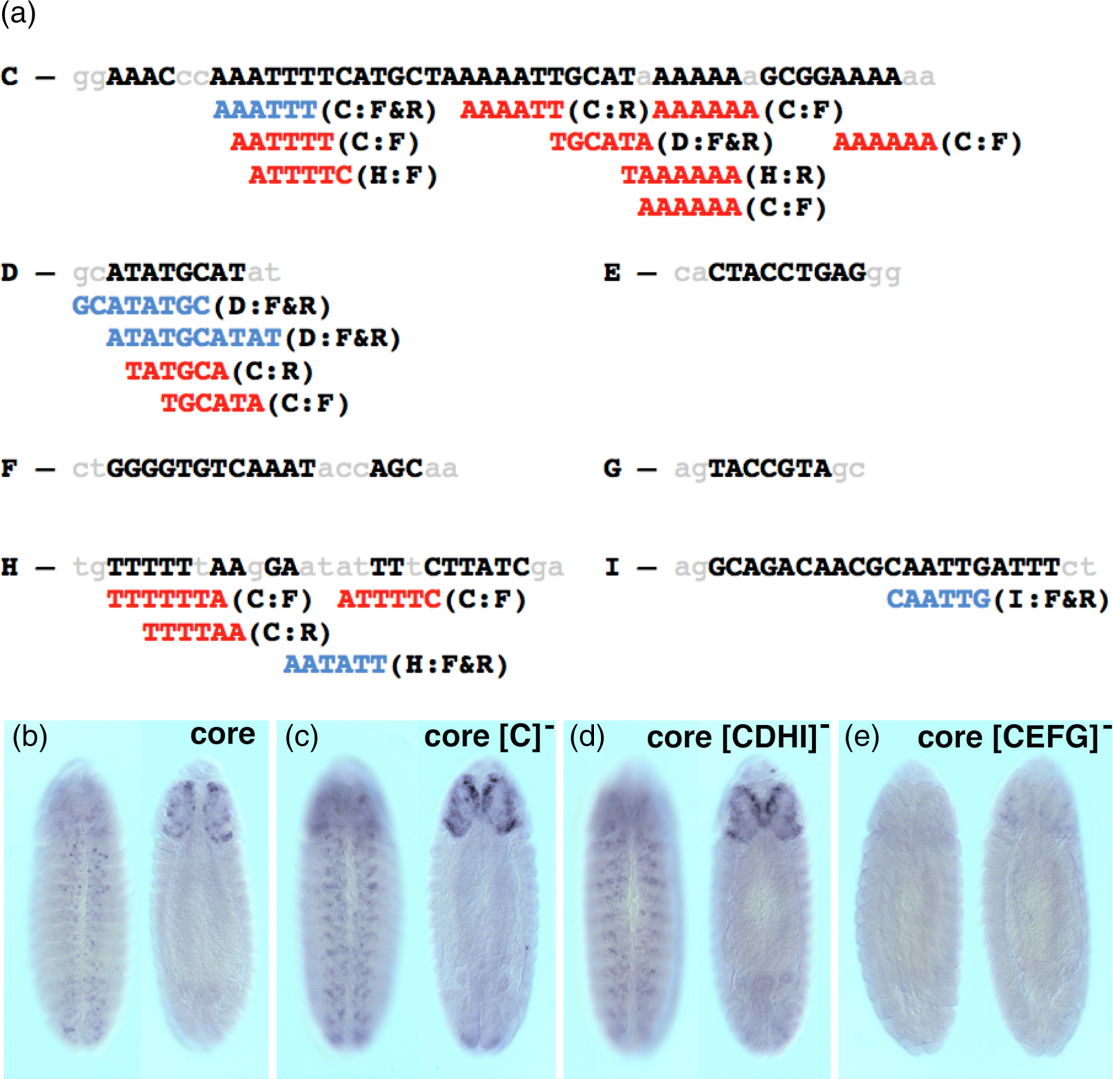
Contribution of *nub‐46* repeat and unique sequence elements within the core enhancer. (a) *cis*‐Decoder analysis of the core CSBs of the *nub‐46* enhancer as defined in Figure 1 reveals that they contain nine repeat and palindromic sequences. Seven of these are found in CSB “C,” three in CSB “D,” four in CSB “H,” one in CSB “I.” CSBs “E,” “F” and “G” contained no repeat elements. The repeat elements are aligned underneath each CSB. Highlighted in red are non‐palindromic repeats and palindromic sequences are highlighted in blue. Following each repeat, in parentheses, is shown the identity of the other CSB that contains this repeat, followed by its forward or reverse orientation represented by the letters F or R. (b–e) Transgene reporter mRNA expression patterns in whole‐mount stage 13 embryos (ventral and dorsal views, anterior up). (b) Reporter expression driven by the *nub‐46* core sequence that contains CSBs “C” through “I.” (c) Reporter expression activated with the core minus CSB “C.” Note, the enhanced expression compared to that achieved with the full core. (d) Reporter expression activated with the core fragment lacking CSB “C” but containing only CSBs “E,” “F,” and “G.” This construct, lacking repeat elements, was sufficient to activate reporter expression. (e) Ventral cord expression was undetectable and only weak reporter expression was detected in the cephalic lobes with the core sequence minus CSB “C” and with repeat‐containing elements “D,” “H,” and “I.”

Given that other *cis*‐regulatory enhancers contain a combination of repeat and unique sequence elements, we hypothesized that *nub‐46* activation may result from a complex set of multiple inputs. Indeed, self‐alignment of conserved sequences within *nub‐46* revealed that the enhancer is made up of 11 distinct repeat and palindromic elements (Figure 4a). Upon closer inspection, we found that seven of the 10 repeat elements are located within the “C” element, and that many of these repeats were also found in CSBs “D,” “H,” and “I” of the enhancer core (Figure 4a).

**Figure 5:**
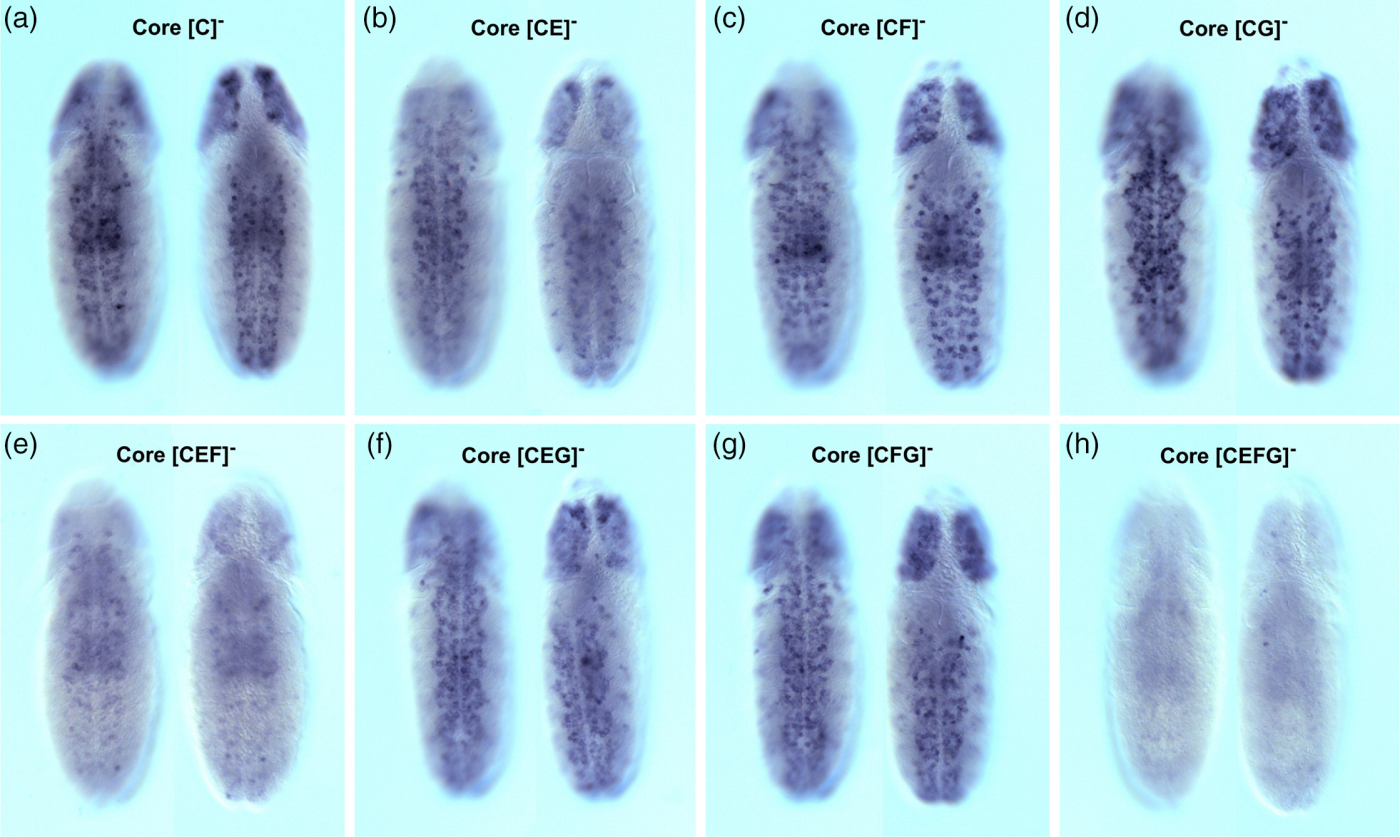
Deletion analysis of the *nub‐46* enhancer core reveals functional overlap among individual conserved sequence blocks. Shown are enhancer CSB transgene reporter mRNA expression patterns in whole‐mount stage 11 embryos (ventral and dorsal views; anterior up). Each of the constructs lacked CSB “C,” containing repressive elements described in Figure 2: (a) *nub‐46* enhancer construct containing CSBs “D,” “E,” “F,” “G,” “H,” and “I” (see Figure 1a for sequence); (b) element minus “E”; (c) element minus “F”; (d) element minus “G”; (e) element minus “E” and “F”; (f) element minus “E” and “G”; (g) element minus “F” and “G”; and (h) element minus “E,” “F,” and “G.” Only loss of both “E” and “F” compromised core activity, while higher enhancer activity was obtained with loss of “F” and “G.”

We next assessed whether the repeat elements within the core are required for enhancer activation. Loss of the “C” element does not significantly affect onset of enhancer‐reporter expression during embryonic VNC development (Figure 4c). Among the six repeats identified within the “C” element, nearly all are present in the “D,” “H,” and “I” elements (Figure 4a), and we speculated that these may compensate for the loss of repeats in the *nub‐46* [C]^−^ mutant. To test this hypothesis, we truncated the core to exclude the “C” element (denoted as the [C^**−**^] in Figure 4c) and then further removed all elements containing repeats (“D,” “H,” and “I” elements) from the core enhancer (referred to as [CDHI]^−^ in Figure 4d). Surprisingly, removal of these CSBs had little or no effect on enhancer activity (Figure 4d). One possible explanation for the lack of any significant effect of element “C” (and other elements containing repeat sequences) on enhancer activation is that activator sequences are located within elements lacking repeats (elements “E,” “F,” and “G”). To investigate whether the “E” (CTACCTGAG), “F” (GGGGTGTCAAATACCAGC), and “G” (TACCGTA) elements are required for enhancer activation, we removed all three elements from the enhancer [CEFG]^−^ and observed that deletion of these resulted in complete loss of reporter activity, suggesting that “E,” “F,” and “G”, containing only unique sequences, are required to activate reporter expression (Figure 4e). To determine whether a subset of these elements is necessary for enhancer function, we tested the effect of different combinations of internal deletions on *cis*‐regulatory activity during embryonic neurogenesis. While removal of either the “E,” “F,” or “G” elements had little or no effect on enhancer function (Figure 5b–d), only the combined loss of “E” and “F” compromised core activity (Figure 5e). Notably, however, we identified increased enhancer activity with loss of “F” and “G” (Figure 5g), whereas loss of all three non‐repeat elements disrupted enhancer function (Figure 5h). Individual deletion of non‐repeat CSBs exhibited minor reduction in enhancer activity within brain lineages (see Figure 5 panels C, D, and G).

**Figure 6:**
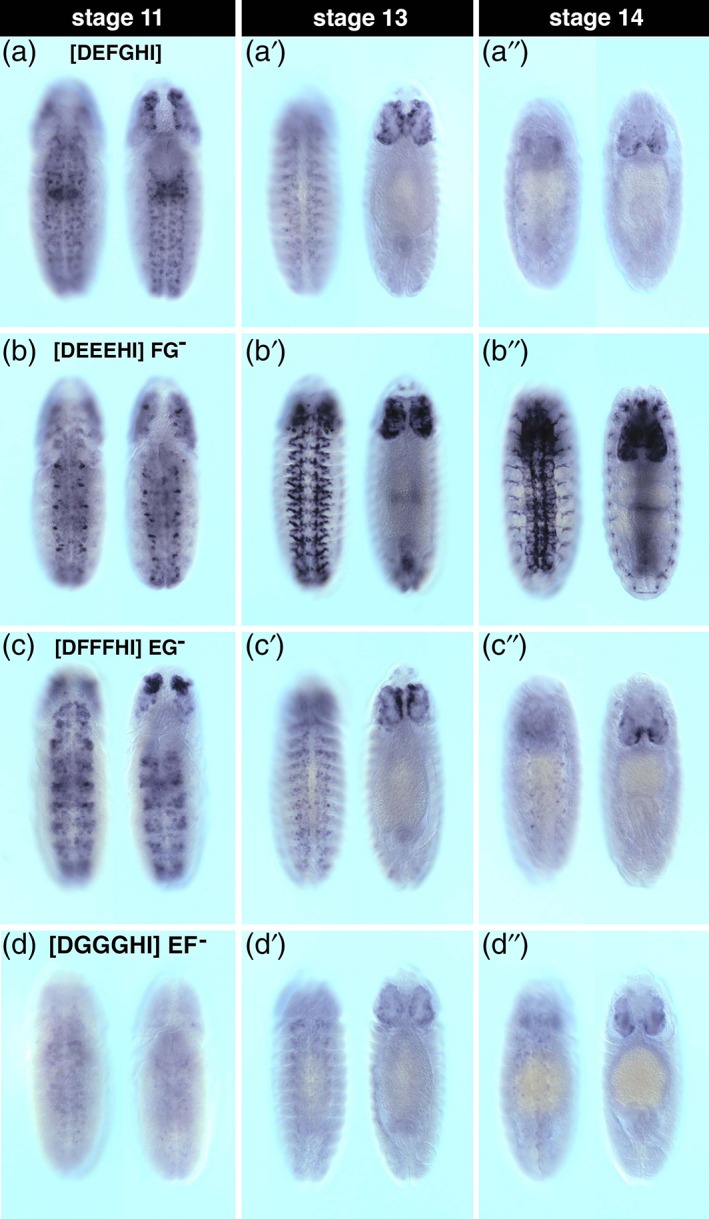
Enhancer function is altered by adding multiple CSBs that lack repeat sequences. Shown are enhancer CSB transgene reporter mRNA expression patterns in whole‐mount stage 11, 13, and 14 embryos (ventral and dorsal views; anterior up). The enhancer constructs lack CSB “C,” which contained repressive elements described in Figure 2, but were engineered to contain triple occurrence of non‐repeat elements “E,” “F,” and “G.” (a) *nub‐46* enhancer construct containing CSBs “D,” “E,” “F,” “G,” “H,” and “I”; (b–d) three copies of the “E” CSB were substituted for the “F” and “G” CSBs; (c) three copies of the “F” CSB were substituted the “E” and “G” CSBs; (d) three copies of the “G” CSB were substituted for the “E” and “F” CSBs (see Figure 1a for sequences)

Given that all three elements lacking repeat sequences are essential for enhancer function, we next asked whether enhancer function is modified by the multiplicity of these sequences. To explore this, we synthesized core enhancers that contain three copies of either element, substituting each into the positions of the other two non‐repeat elements. We also examined construct expression during multiple stages of CNS development (Figure 6). When we replaced the “F” and “G” elements with “E” elements (Figure 6b), increasing the number of “E” elements to three, higher enhancer activity were observed within subsets of NBs compared to the wild‐type during stage 11 (Figure 6a,b). However, by stage 13, we observed higher levels of enhancer activity throughout the CNS (Figure 6b’). Notably, we also observed ectopic expression within putative PNS lineages during stage 14 (Figure 6b”). It should be noted that additional co‐localization experiments using cell lineage markers would be needed to substantiate the ectopic expression. Increasing the number of “F” elements also altered core enhancer activity, but the effect was limited to a subset of lateral VNC NBs and dorso‐anterior cephalic lobe cells during early stage 12. These differences were not apparent at stage 13 and stage 14 (Figure 6c). Increasing the number of “G” elements resulted in diminished expression at all three stages examined (Figure 6d).

## 4 SUMMARY

The principal findings of this study are the identification of a core sequence within the *nub‐46* NB enhancer that is sufficient to recapitulate the embryonic expression pattern of *nubbin* and that novel non‐repeated conserved sequences are required for enhancer activity. Our study has delimited the target of Cas repression to a CSB containing two adjacent 9‐mer sequences corresponding to the TF DNA‐binding motif for Cas in CSB “C.” Nevertheless, the possibility still exists that Cas is not the only repressor of *nub‐46* during embryonic CNS development.

We have also localized activator CSBs that contain uniquely represented sequences within the enhancer, suggesting that the enhancer may be regulated by as yet uncharacterized TF activators that play a role in the temporal regulation of *nubbin*. Our data suggests that multiple copies of either “E” or “F” can function as an activator within the enhancer core. While previous studies have suggested that clusters of repeat regulatory sequences are an important aspect of enhancer regulation (Brody et al., 2012; Gotea et al., 2010; Lifanov, Makeev, Nazina, & Papatsenko, 2003; reviewed by Taher, 2013), this study points to unique non‐repeated motifs as targets of transcriptional activators. While our initial observations revealed altered expression outside the spatial/temporal boundaries of *nub‐46* activity, further experiments using cell‐type specific markers are needed to confirm this ectopic expression.
